# Characterization of Cep85 – a new antagonist of Nek2A that is involved in the regulation of centrosome disjunction

**DOI:** 10.1242/jcs.171637

**Published:** 2015-09-01

**Authors:** Canhe Chen, Fang Tian, Lin Lu, Yun Wang, Zhe Xiao, Chengtao Yu, Xianwen Yu

**Affiliations:** State Key Laboratory of Cellular Stress Biology, School of Life Sciences, Xiamen University, Xiamen, Fujian 361102, China

**Keywords:** Cep85, Nek2A, Centrosome disjunction

## Abstract

Nek2 has been implicated in centrosome disjunction at the onset of mitosis to promote bipolar spindle formation, and hyperactivation of Nek2 leads to the premature centrosome separation. Its activity, therefore, needs to be strictly regulated. In this study, we report that Cep85, an uncharacterized centrosomal protein, acts as a binding partner of Nek2A. It colocalizes with isoform A of Nek2 (Nek2A) at centrosomes and forms a granule meshwork enveloping the proximal ends of centrioles. Opposite to the effects of Nek2A, overexpression of Cep85 in conjunction with inhibition of the motor protein Eg5 (also known as KIF11) leads to the failure of centrosome disjunction. By contrast, depletion of Cep85 results in the precocious centrosome separation. We also define the Nek2A binding and centrosome localization domains within Cep85. Although the Nek2A-binding domain alone is sufficient to inhibit Nek2A kinase activity *in vitro*, both domains are indispensable for full suppression of centrosome disjunction in cells. Thus, we propose that Cep85 is a bona fide Nek2A-binding partner that surrounds the proximal ends of centrioles where it cooperates with PP1γ (also known as PPP1CC) to antagonize Nek2A activity in order to maintain the centrosome integrity in interphase in mammalian cells.

## Abstract (precis)

**Summary:** Cep85 acts as a binding partner of Nek2A to prevent premature centrosome separation in interphase by inhibiting Nek2A activity.

## INTRODUCTION

The centrosome is an organelle that serves as the main microtubule-organizing center (MTOC) to promote bipolar spindle formation and timely mitotic progression (Bornens, 2002; Nigg and Raff, 2009). It consists of a pair of centrioles and a surrounding protein meshwork called pericentriolar material (Bornens et al., 1987). Highly reminiscent of DNA replication, the centrosome duplicates once per cell cycle (Nigg, 2006; Tsou and Stearns, 2006). Following cytokinesis, a single centrosome is replicated during S phase, separated in G2/M phase and pulled to the spindle poles at the onset of mitosis (Bettencourt-Dias and Glover, 2007; Mardin and Schiebel, 2012). In interphase, the two centrioles in one centrosome are connected by a proteinaceous linker comprising centrosomal Nek2-associated protein 1 (C-Nap1, also known as CEP250), rootletin and LRRC45 (Bahe et al., 2005; Fry et al., 1998a, 1995; He et al., 2013). C-Nap1 localizes at the proximal ends of the centriole and is considered to act as a docking site for rootletin and LRRC45. Both rootletin and LRRC45 interact and form a fiber-like structure to link the pair of centrioles (He et al., 2013). This intercentriole linkage needs to be dissolved before mitosis to allow bipolar spindle formation. Multiple kinases have been demonstrated to be involved in this procedure, including cyclin B2 and cyclin-dependent kinase 1 (Cdk1) (Nam and van Deursen, 2014), Aurora-A (Pugacheva and Golemis, 2005; Zhou et al., 1998), Polo-like kinases (Plks) (Glover et al., 1998; Mardin et al., 2011), and NIMA (never in mitosis A)-related kinase 2 (Nek2) (Fry et al., 1998b). Among them, Nek2A has been shown to be the main kinase directly responsible for the phosphorylation and dissolution of the linker proteins.

Nek2, a member of the NIMA-related kinase (Nek) family of serine/threonine protein kinases, has three splicing isoforms, Nek2A, Nek2B and Nek2C (Fry, 2002; O'Regan et al., 2007). Depending on whether there is a nuclear localization sequence on their C-termini, they are differentially distributed in cells. Nek2A is located within the nucleus and cytoplasm, Nek2B is mainly cytoplasmic, and Nek2C is predominantly nuclear (Hames and Fry, 2002; O'Regan et al., 2007). Interestingly, Nek2A and Nek2B exhibit distinct cell-cycle-dependent expression patterns. Both are very low in G1, rise significantly at the G1/S transition and remain high throughout S and G2 phase (Hames and Fry, 2002; Hames et al., 2001; Hayes et al., 2006). Unlike Nek2B, which remains at about the same level as in G2, Nek2A is degraded rapidly at the onset of mitosis owing to anaphase promoting complex (APC)-mediated ubiquitylation (Hames et al., 2001; Hayes et al., 2006). Consistent with this, Nek2A is not required for mitotic entry in human cells, but does function at the G2/M transition to phosphorylate the linker proteins, such as C-Nap1, rootletin and LRRC45 (He et al., 2013; Mardin and Schiebel, 2012). Phosphorylation leads to dissolution of linker and, eventually, separation of centrosomes. It is well known that hyperactivation of Nek2 results in the premature separation of centrosomes in interphase (Faragher and Fry, 2003; Fry et al., 1998b; Mardin and Schiebel, 2012). As Nek2A protein levels remain high in the S to G2 phase, there must be some mechanisms by which Nek2A activity at centrosomes remains low in interphase to prevent centrosome disjunction but high at the G2/M transition to promote centrosome disjunction.

Nek2A activity at the centrosome is subject to both positive and negative regulation. At the G2/M transition, Hippo pathway components and cell-cycle-dependent kinase Plk1 have been reported to be involved in the elevation of Nek2A activity at centrosomes. Two Hippo pathway components, the mammalian sterile 20-like kinase 2 (Mst2, also known as STK3) and the scaffold protein Salvador (Sav1), directly interact with Nek2A and enhance its centrosomal accumulation so that it can phosphorylate C-Nap1 and rootletin, thereby facilitating centrosome disjunction in conjunction with the kinesin-5 motor protein Eg5 (also known as KIF11) (Mardin et al., 2010). Meanwhile, as Plk1 increases its activity at G2 (Golsteyn et al., 1995), it phosphorylates Mst2 in the Mst2–Nek2A–PP1γ complex at centrosomes, leading to a reduction in the amount of PP1γ (also known as PPP1CC) in the complex (Mardin et al., 2011). This in turn increases the localized Nek2A activity to promote centrosome separation. In interphase, protein phosphatase 1 (PP1), including PP1α (also known as PPP1CA) and PP1γ (Meraldi and Nigg, 2001; Mi et al., 2007), the focal adhesion scaffolding protein HEF1 (also known as NEDD9) (Pugacheva and Golemis, 2005) and pericentrin (also called kendrin) (Matsuo et al., 2010) have been found to negatively regulate Nek2 activity. PP1 binds directly to a KVHF motif in the non-catalytic C-terminal region of Nek2A (Eto et al., 2002; Helps et al., 2000). Although whether the reduction of Nek2A activity caused by the PP1-mediated decrease in Nek2A autophosphorylation also contributes to the maintenance of centrosome integrity in interphase needs to be clarified, it is widely accepted that PP1γ at centrosomes antagonizes Nek2A activity by playing direct roles in the dephosphorylation of the linker proteins that are phosphorylated by Nek2A (Mardin et al., 2011). HEF1 inhibits Nek2A activity and prevents its accumulation at centrosomes (Pugacheva and Golemis, 2005), and pericentrin associates with Nek2A kinase and inhibits its kinase activity (Matsuo et al., 2010). However, it is not yet clear how the localized Nek2A activity at the proximal ends of centrioles is regulated. In this study, we reveal that Cep85 interacts with Nek2A, localizes to the proximal ends of the centrioles and functions as a negative regulator of Nek2A to maintain the centrosome integrity in interphase.

## RESULTS

### Identification of Cep85 protein as a binding partner of Nek2A

**Fig. 1.**
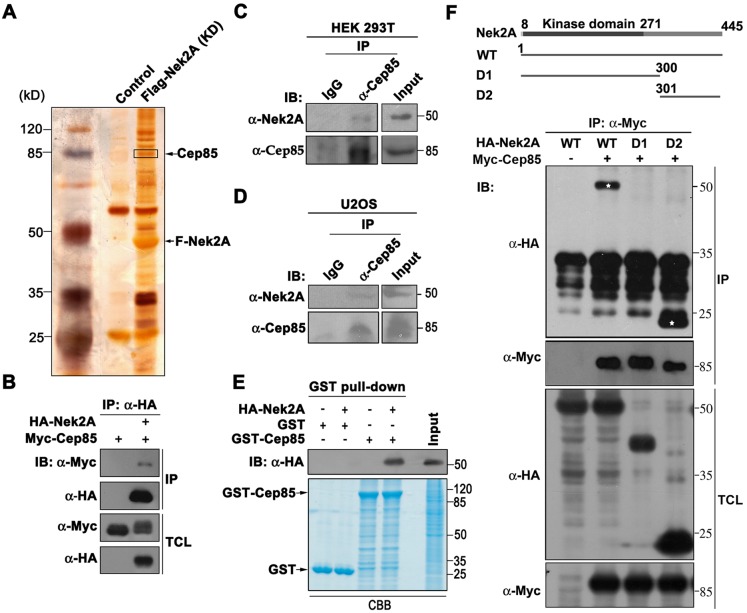
**Identification of Cep85 as a binding partner of Nek2A.** (A) Flag-tagged kinase-dead (KD) Nek2A was expressed in HEK293T cells, affinity purified with M2 agarose and separated by SDS-PAGE, followed by silver staining to visualize the locations of individual proteins. The protein bands around 85 kDa inside the boxed area were identified by mass spectrometry to contain the Cep85 protein. (B) Myc–Cep85, co-expressed with HA–Nek2A in HEK293T cells, was immunoprecipitated with anti-Myc antibody. HA–Nek2A in the immunoprecipitate (IP) and total cell lysates (TCL) was visualized with anti-HA antibody by western blotting (IB). (C) The endogenous Cep85 in HEK293T cell lysate was immunoprecipitated with anti-Cep85 antibody and anti-Nek2A antibody was used to detect Nek2A in the precipitate. (D) The same experiment as in C was performed using U2OS cell lysates. (E) GST pulldown assays were carried out with GST–Cep85 protein expressed in and purified from *E. coli*, and HA–Nek2A overexpressed in HEK293T cells. CBB, Coomassie Brilliant Blue. (F) A schematic of Nek2A and its truncated mutants is shown in the upper panel. Myc–Cep85 and HA–Nek2A constructs were co-expressed in HEK293T cells. The binding region in Nek2A to Cep85 defined by co-immunoprecipitation and western blot analysis is shown in the lower panel. Asterisks indicate the positions of Nek2A proteins.

Nek2A expression levels remain high in the S to G2 phase, however, its activity at the proximal ends of centrioles needs to be kept low to ensure that the centrosome is not to be separated precociously (Mardin and Schiebel, 2012). In order to identify potential Nek2A-binding partners that might suppress Nek2A activity, we expressed a Flag-tagged kinase-dead mutant Nek2A in human embryonic kidney 293T (HEK293T) cells and used M2 agarose to affinity purify Flag–Nek2A, followed by mass spectrometry analysis to determine the identities of Nek2A-binding proteins (Fig. 1A). Among the identified proteins, centrosomal protein 85 kDa (Cep85), also known as coiled-coil domain-containing protein 21 (CCDC21), was a particular interesting candidate. It was initially identified with other 21 proteins as new centrosomal components by complementary proteomics strategies (Jakobsen et al., 2011). Except for its spindle pole association, to date, no other studies have been performed. Human Cep85 was predicted to contain two coiled-coil domains (supplementary material Fig. S1A). Its orthologs were well conserved in many eukaryotes, including *Mus musculus*, *Xenopus*, *Danio rerio* and *Gallus gallus* (supplementary material Fig. S1B).

In order to further characterize Cep85, we raised a rabbit polyclonal antibody against Cep85. A recombinant GST fusion protein containing the C-terminal region, corresponding to amino acids 544–762 of human Cep85, was used as an antigen and the produced crude sera were purified by affinity immunoabsorption. This purified antibody could specifically recognize those proteins carrying the C-terminal portion of Cep85 including the wild-type (WT) protein and amino acids 544–762 (supplementary material Fig. S1C) and could specifically immunoprecipitate Myc–Cep85 protein overexpressed in HEK293T cells (supplementary material Fig. S1D). The specificity of this antibody to the endogenous Cep85 protein was also validated in an experiment with small interfering RNA (siRNA)-mediated Cep85 depletion (see Fig. 6A–C).

To verify the interaction between Cep85 and Nek2A, we first co-expressed Myc-tagged Cep85 and HA-tagged Nek2A in HEK293T cells and ascertained that Cep85 could be coimmunoprecipitated by Nek2A (Fig. 1B). We also noticed that Nek2A, once co-expressed with Cep85, caused a mobility shift in the Cep85 protein, suggesting that Cep85 might be phosphorylated by Nek2A. We also immunoprecipitated endogenous Cep85 protein from HEK293T cell lysates and found that Nek2A indeed coimmunoprecipitated with Cep85 (Fig. 1C). The same result was obtained when using U2OS cell lysates (Fig. 1D), suggesting that endogenous Cep85 and Nek2A proteins physically interact and form a complex in cells. Furthermore, an *in vitro* GST pulldown assay revealed that a bacterially produced GST fusion Cep85 protein could specifically pull down HA-tagged Nek2A protein produced in HEK293T cells (Fig. 1E). These results confirm that Cep85 is a binding partner of Nek2A in cells. To define the Cep85-binding region in Nek2A, we generated two truncated mutants, D1 and D2, and performed coimmunoprecipitation analysis (Fig. 1F). We found that Cep85 could bind to WT Nek2A and the C-terminal region (D2) of Nek2A but not to its N-terminal region (D1), which harbors the Nek2A kinase domain.

### Cep85 is a ubiquitous, stable protein that accumulates at centrosomes through the centrosome localization domain

**Fig. 2.**
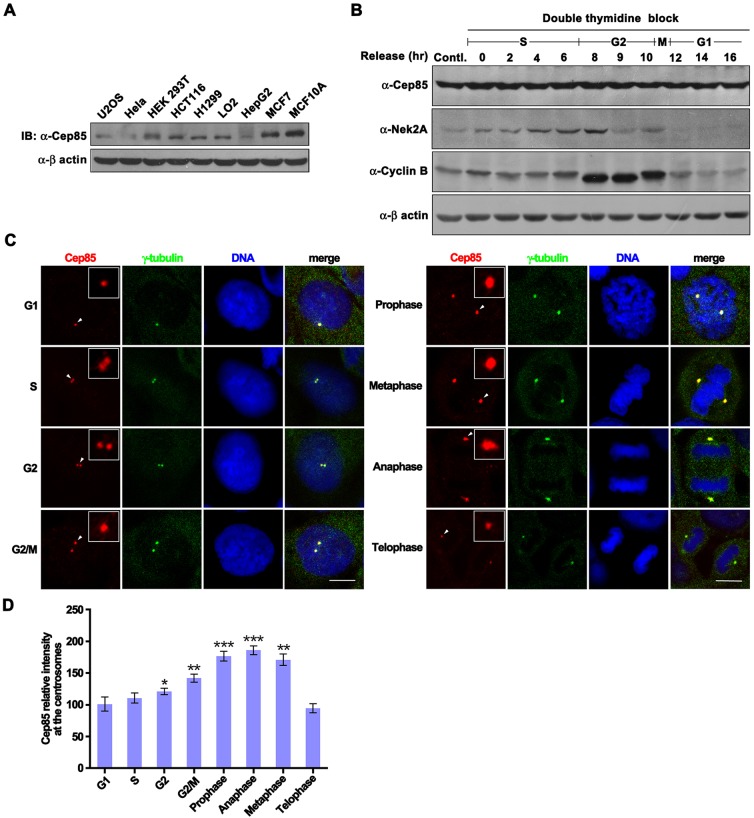
**Cep85 expression levels in cell lines and at different stages of the cell cycle, and its cell-cycle-dependent localization at centrosomes.** (A) The cell lysates from indicated cell lines were prepared and subjected to western blotting (IB). (B) HeLa cells were synchronized at G1/S using a double thymidine block. After releasing, cells were harvested at indicated time points prior to western blot analysis with antibodies to detect the endogenous levels of individual proteins. (C) U2OS cells were fixed and immunostained with antibodies against endogenous Cep85 (red) and γ-tubulin (green). Arrowheads point to Cep85 protein at centrosomes, which is enlarged and shown in the inset on individual images. Scale bars: 5 µm. (D) The intensity of Cep85 signal at centrosomes was quantified. Data are mean±s.e.m. Three independent experiments were performed, and 20 cells were analyzed for each experiment. *P<0.05; **P<0.01; ***P<0.001 versus G1.

Taking advantage of the fact that our antibody can specifically detect Cep85 protein at an endogenous level, we decided to determine Cep85 protein levels in various cell lines. We found that Cep85 was ubiquitously expressed in all cell lines collected although differential expression levels were detected. High levels of Cep85 were found in the HEK293T, MCF7 and MCF10A cell lines, medium levels in the HCT116, LO2 and H1299 cell lines, and low levels in U2OS, HeLa and HepG2 cells (Fig. 2A). The dependency of Cep85 protein levels on the cell cycle was also examined. HeLa cells were synchronized at the G1/S boundary by a double thymidine block and then released into the cell cycle by incubating with thymidine-free fresh medium. Cells were harvested at different time intervals and subjected to western blot analysis. Consistent with previous reports (Hames and Fry, 2002), Nek2A levels remained low in M and G1 phase but were high in S and G2 phase; cyclin B tremendously increased levels at late G2 and early M phase. In contrast, Cep85 levels remained nearly unchanged throughout the cell cycle (Fig. 2B).

To examine the subcellular localization of Cep85 protein, we utilized the anti-Cep85 antibody to immunostain endogenous Cep85 and anti-γ-tubulin antibody to mark centrosomes. Fluorescent microscopy was used to trace the subcellular localization of Cep85 protein at different stages of cell cycles. Cep85 was found to form small granules that were ubiquitously scattered in the cytoplasm at low levels but were not present in the nucleus in interphase (Fig. 2C). With the assistance of γ-tubulin staining, we observed that Cep85 was localized to the centrosomes at all stages of cell cycle with distinctive levels. It was localized to centrosomes at a low level in G1 phase and a slightly increased level in S phase, with gradually elevated levels being observed during G2 phase (Fig. 2C,D). The levels of Cep85 at centrosomes were further increased at G2/M, reached a peak at spindle poles at early mitotic stages and remained high until the end of anaphase (Fig. 2C,D). These results reveal that Cep85 is indeed a centrosome component and suggest that the centrosomal association of Cep85 might be subject to regulation.

**Fig. 3.**
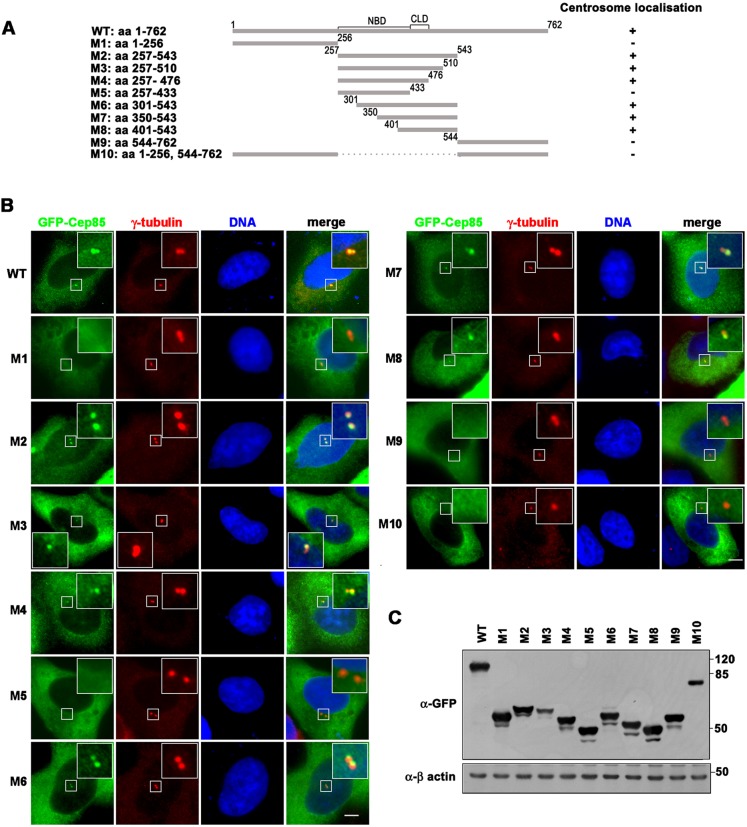
**The centrosome localization domain in Cep85 is required for its centrosome association.** (A) A schematic of Cep85 and its truncated mutants is shown. The numbers indicate the positions of the first or the last amino acid of individual fragments. +, positive; −, negative for centrosome localization. (B) U2OS cells expressing individual GFP–Cep85 proteins were immunostained with antibody against γ tubulin (red) to indicate centrosomes. Boxed areas are enlarged in the insets on individual images. Scale bars: 5 µm. (C) The expression levels of individual GFP–Cep85 proteins in U2OS cells are shown by western blotting with anti-GFP antibody.

To determine the domain mediating Cep85 centrosome localization, we generated a series of GFP-tagged truncated mutants and transiently overexpressed them in U2OS cells (Fig. 3A,C). We observed that WT Cep85 as well as those mutants harboring amino acids 434–476, including M2, M3, M4, M6, M7 and M8, located to the centrosomes marked by γ-tubulin immunostaining (Fig. 3B). In contrast, the other mutants not carrying amino acids 434–476, including M1, M5, M9 and M10, did not localize to centrosomes (Fig. 3B). Therefore, the region 434–476 in Cep85 is required for its centrosomal localization.

### Cep85 colocalizes with Nek2A and forms a granule meshwork enveloping the proximal ends of centrosomes

**Fig. 4.**
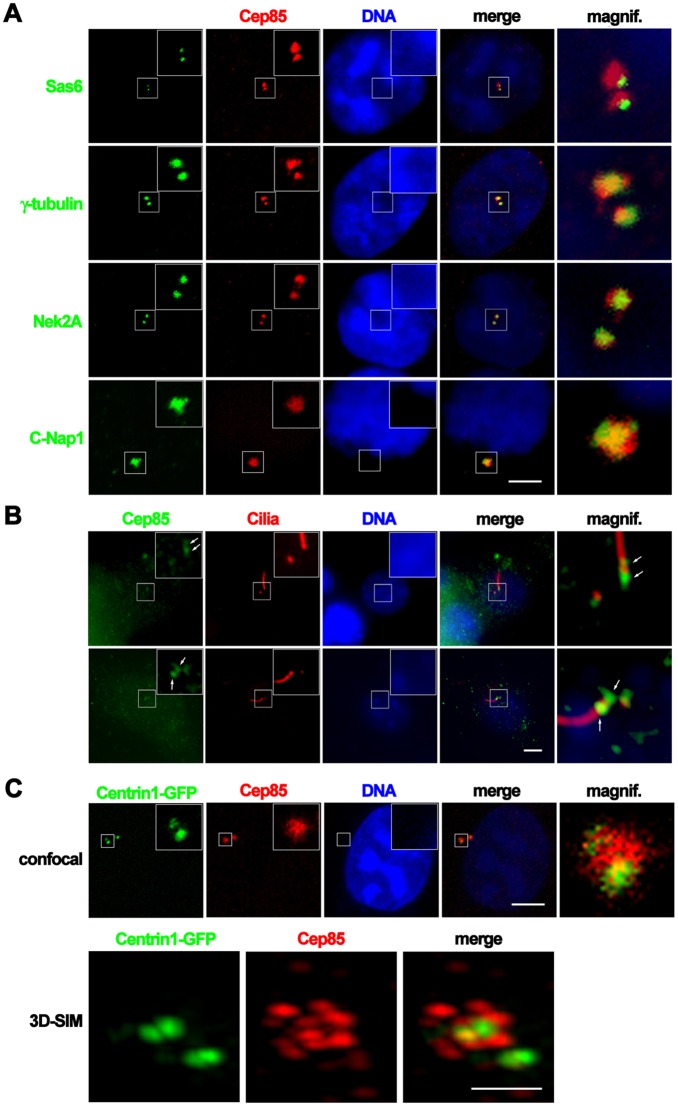
**Cep85 protein is a pericentriolar material component and colocalizes with Nek2A at the proximal ends of the centrioles.** (A) Immunofluorescence of Cep85 (red), Sas-6 (green), γ-tubulin (green), Nek2A (green) and C-Nap1 (green) is presented. Boxed areas are enlarged in the insets. A higher magnification image of the boxed area in individual merged images is shown on the right. Scale bar: 5 µm. (B) NIH3T3 cells were serum-starved for 24 h prior to immunostaining with anti-acetylated-tubulin antibody, to mark the primary cilia and daughter centriole (red), and anti-Cep85 antibody (green). Arrows highlight the proximal and distal localizations of Cep85 protein at the mother centriole. Boxed areas are enlarged in the insets. A higher magnification image of the boxed area in individual merged images is shown on the right. Scale bar: 5 µm. (C) The stable centrin1–GFP U2OS cells were fixed and immunostained with anti-Cep85 antibody. The confocal microscopic images are shown in the upper panel. An enlarged view of the boxed area is shown in the inset. The magnified view of the boxed centrosome in the merged image is illustrated on the right. Scale bar: 5 µm. The lower panels show a 3D reconstruction of SIM images of a U2OS cell at early G2 phase with centrin1–GFP to mark centrioles (green) and anti-Cep85 antibody to mark the structure of Cep85 (red) surrounding the centrioles. Scale bar: 1 µm.

The immunostaining assays clearly demonstrated that Cep85 is a centrosome-associated protein. To further investigate the localization and structure of Cep85 protein at centrosomes, we carried out fluorescent microscopy analysis in detail. We first co-stained U2OS cells with antibodies against Cep85 and Sas-6 (Leidel et al., 2005), a central scaffolding component of the daughter centriole. Cep85 did not colocalize with Sas-6, indicating that Cep85 is not a procentriole component (Fig. 4A). It partially overlapped with γ-tubulin suggesting that Cep85 is a pericentrosomal protein (Fig. 4A). When co-stained with Nek2A, Cep85 displayed extensive colocalization with Nek2A in interphase, suggesting that Cep85 might locate to the same locations as Nek2A at centrosomes (Fig. 4A).

Nek2A has previously been found to localize to the proximal and distal ends of centrioles (Fry et al., 1998a; Spalluto et al., 2012). We were curious to see whether its interacting protein Cep85 also localized to the ends of centrioles*.* NIH3T3 cells were serum-starved to induce primary cilia formation, followed by co-staining with anti-Cep85 antibody and anti-acetylated-tubulin antibody to mark the cilia and centrioles. We unambiguously found that Cep85 proteins indeed formed three discrete patches (Fig. 4B). Two of them localized to and enveloped the proximal ends of both mother basal body and daughter centriole, and the third surrounded the boundary between the basal body and axoneme (Fig. 4B). The proximal end localization of Cep85 was further confirmed by the observation that Cep85 was colocalized with C-Nap1 (Fig. 4A), which is known to associate specifically with the proximal ends of centrioles as revealed by immunoelectron microscopy (Fry et al., 1998a). These results suggest that Cep85 and Nek2A colocalize at the proximal ends of the centrioles. To better visualize the Cep85 structure, we generated a U2OS cell line stably expressing centrin1–GFP to mark the centrioles and single immunostained with anti-Cep85 antibody. Confocal microscopy revealed that Cep85 formed a granule meshwork surrounding centrin during interphase (Fig. 4C). This structure appeared to be more clear when observed with three-dimensional structured illumination microscopy (3D-SIM) (Fig. 4C), which shows that it underwent considerable expansion in mitosis (supplementary material Fig. S2). Thus, these data suggest that Cep85 is a pericentriolar material protein and forms a granule meshwork encasing the proximal ends of both mother and daughter centrioles.

### Up- and down-regulation of Cep85 expression causes centrosome abnormalities that are exactly the opposite to the effects of Nek2A

Nek2A has been shown to be an important kinase responsible for the centrosome disjunction at G2/M by phosphorylating linker proteins such as C-Nap1 and rootletin (Fry, 2002; Mardin and Schiebel, 2012). Previous studies have demonstrated that overexpression of Nek2A leads to premature separation of centrosomes, and depletion of Nek2A in conjunction with inhibition of Eg5 activity inhibits the disjunction of centrosome during mitosis (Fry et al., 1998b; Mardin et al., 2010). Given that Cep85 was identified as a Nek2A-interacting protein and colocalized with Nek2A at centrosomes, we reasoned that alteration of Cep85 protein levels might cause centrosome abnormalities by altering Nek2A in cells. We therefore established two systems to determine the functional significance of Cep85.

**Fig. 5.**
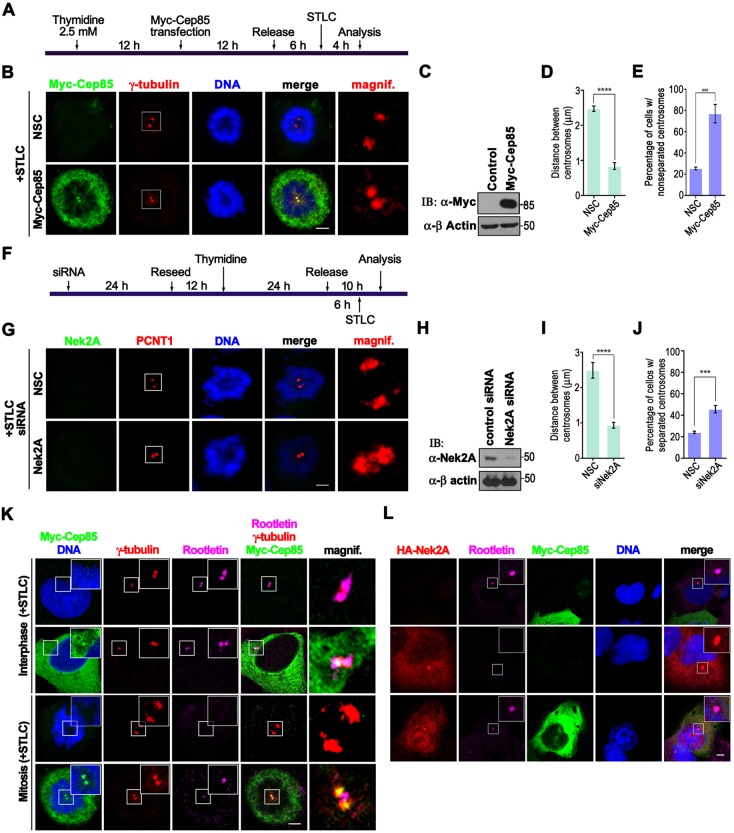
**Overexpression of Cep85 inhibits centrosome disjunction, similar to the effect of Nek2A depletion, and prevents the rootletin release from centrosomes caused by Nek2A overexpression.** (A) Schematic outline of experiments shown in B–E. U2OS cells overexpressing Myc–Cep85 were enriched at G1/S by a single thymidine block followed by release and treatment with STLC to trap cells in prometaphase. (B) Cells were fixed and stained to visualize Myc–Cep85 (green), γ-tubulin (red) and DNA (blue) by immunofluorescence. (C) The expression levels of Myc–Cep85 in U2OS cells as demonstrated by western blotting (IB). (D) The distance between two centrosomes of cells in B. ****P<0.0001. (E) The histogram indicates the percentage of cells with nonseparated centrosomes. Centrosomes were counted as separation when the distance between two centrioles was over 1 μm. ***P<0.001. (F) Schematic outline of experiments shown in G–J. U2OS cells transfected with Nek2A siRNA or control siRNA were enriched at G1/S by a single thymidine block and treated with STLC to trap cells in prometaphase. (G) Cells were fixed and stained with an antibody against Nek2A (green) and PCTN1 (red) to indicate the centrosomes. (H) The efficiency of siRNA to deplete Nek2A proteins is shown. Unsynchronized U2OS cells transfected with indicated siRNAs were maintained for 70 h prior to western blot analysis. (I) The distance between two centrosomes for cells in G. ****P<0.0001. (J) The histogram indicates the percentage of cells with nonseparated centrosomes. ***P<0.001. (K) Immunofluorescence analysis of rootletin in Cep85-overexpressing cells. U2OS cells were treated according to the procedure shown in A. Cells were fixed and immunostained with antibodies to detect Myc–Cep85 (green), γ tubulin (red) and rootletin (purple). The enlarged view of the boxed area is shown in the insets. The magnified view of the boxed centrosome in the merged image is illustrated on the right. (L) Cells overexpressing either HA–Nek2A or both HA–Nek2A and Myc–Cep85 were fixed and immunostained with anti-HA antibody to detect HA–Nek2A (red), anti-Myc antibody to detect Myc–Cep85 (green), and anti-rootletin antibody (purple). An enlarged view of the boxed area is shown in the inset. In B, G, K and L, the enlarged view of the boxed area is shown in the inset on individual image; the magnified view of the boxed centrosome in the merged image is illustrated on the right except for in L. Scale bars: 5 µm. In D, E, I and J, three independent experiments were performed with 30 cells were analyzed for each experiment; data are mean±s.e.m. NSC, non-specific control.

In order to ascertain whether upregulation of Cep85 expression levels would affect centrosome disjunction, we overexpressed Cep85 in U2OS cells and synchronized them at G1/S prior to releasing into M phase (supplementary material Fig. S3A). In the absence of treatment with STLC, an inhibitor of Eg5, Cep85-expressing cells did not arrest at any specific cell cycle stages within 22 h; centrosomes could normally separate and migrate to form bipolar spindle poles exactly like the control cells (supplementary material Fig. S3B). When treated with STLC (Fig. 5A), synchronized cells often arrested at prometaphase. In ∼80% of the control cells with no Cep85 overexpression, the centrosome disjunction occurred normally, and the distance between two centrosomes was over 2 µm (Fig. 5B–E). In contrast, the two centrosomes in over 75% of Cep85-overexpressing cells remained in close proximity at a distance of less than 1 µm (Fig. 5B–E). These results suggest that overexpression of Cep85 can prevent centrosome disjunction. We further confirmed this conclusion by performing rootletin immunostaining. During interphase, the linker protein rootletin remained at the centrosomes in both Cep85-overexpressing and non-overexpressing cells (Fig. 5K). During mitosis, rootletin still remained between two nonseparated centrosomes in Cep85-overexpressing cells; however, it completely disappeared in the control cells (Fig. 5K). This phenomenon was exactly like those cells treated with siRNA to deplete Nek2A protein in cells (Fig. 5F–J), as also shown previously (Mardin et al., 2010), suggesting that Cep85 might counteract Nek2A activity. If this is true, then overexpressing Cep85 would alleviate effects of Nek2A overexpression on centrosomes. To verify this hypothesis, we performed rootletin immunostaining analysis. In contrast to the control cells, which retain rootletin at the centrosome in interphase, overexpressing Nek2A resulted in complete disappearance of rootletin at centrosomes. Remarkably, once Cep85 was co-expressed with Nek2A, the rootletin signal reappeared at centrosomes (Fig. 5L), indicating that Cep85 can antagonize Nek2A activity.

**Fig. 6.**
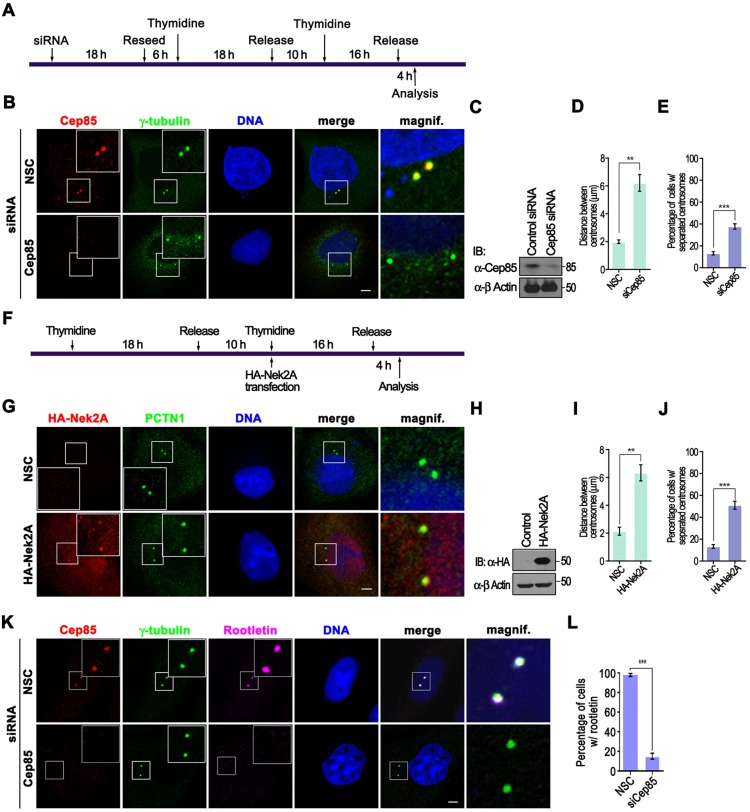
**Depletion of Cep85 results in centrosome splitting, similar to the effect of Nek2A overexpression, and disappearance of the rootletin signal at centrosomes in G2.** (A) Schematic outline of experiments shown in B–E. HeLa cells were transfected with siRNA followed by a double thymidine block and release at G2 phase prior to analysis. (B) Cells were fixed and immunostained with antibodies against Cep85 (green) and γ-tubulin (red). (C) The efficiency of siRNA to deplete endogenous Cep85 is shown by western blotting (IB). (D) The histogram indicates the distance between centrosomes. **P<0.01. (E) The histogram indicates the percentage of cells with separated centrosomes. A centrosome distance exceeding 2 µm was counted as separated. ***P<0.001. (F) Schematic outline of experiments shown in G–J. HeLa cells were subjected to a double thymidine block and release to enrich them at G1/S. HA–Nek2A was transfected into the cells at the indicated time point, 20 h prior to analysis. (G) Cells were fixed and subjected to immunostaining with antibodies to stain HA–Nek2A (red) and γ-tubulin (green). (H) The expression levels of HA–Nek2A in cells are shown by western blotting. (I) The histogram indicates the distance between centrosomes. **P<0.01. (J) The histogram indicates the percentage of cells with separated centrosomes. Centrosome distance exceeding 2 µm was counted as separated. ***P<0.001. (K) Immunofluorescence analysis of rootletin in Cep85-depleted cells. U2OS cells were treated according to the procedure shown in A. Cells were fixed and immunostained with antibodies against Cep85 (red), γ-tubulin (green) and rootletin (purple). (L) The histogram indicates the percentage of cells lacking rootletin signal at centrosomes in G2 after Cep85 depletion. ***P<0.001. In B, G and K, the enlarged view of the boxed area is shown in the inset on individual image, and the magnified view of the boxed centrosome in the merged image is illustrated on the right. Scale bars: 5 µm. In D, E, I, J and L, results are from three independent experiments with 30 cells were analyzed for each experiment; data are mean±s.e.m. NSC, non specific control siRNA.

Given that upregulation of Cep85 can inhibit Nek2A activity, we reasoned that reducing Cep85 protein levels would lead to an enhancement of the activity of Nek2 and premature centrosome separation. We found that is indeed the case as ∼40% of those cells with no detectable level of Cep85, which was depleted by siRNA, displayed premature separation of centrosomes (Fig. 6B–E). This was very similar to the effect of Nek2A overexpression that caused splitting of centrosomes in ∼50% of cells (Fig. 6F–J). We also observed that over 80% of cells with non-detectable level of Cep85 completely lost rootletin at centrosomes in G2/M phase (Fig. 6K,L). Thus Cep85 might play some roles that are opposite to Nek2A in maintenance of centrosome integrity in the S and G2 phases. However, this might simply occur through altering the centrosome structure or accumulation of Nek2A at centrosomes. To rule out these possibilities, we examined the localization and intensity of γ-tubulin and Nek2A after both up- or down-regulation of Cep85. We found that neither up- nor down-regulation of Cep85 changed the intensity or centrosome localization of γ-tubulin and Nek2A, although centrosome splitting revealed by staining either Nek2A or γ-tubulin was easily detected in those Cep85-depleted cells (supplementary material Fig. S4). Thus, both experiments with up- and down-regulation of Cep85 expression indicate that Cep85 antagonizes Nek2A activity in cells.

### Cep85 inhibits Nek2A kinase activity, and the Cep85 region containing amino acids 257–433 is sufficient to bind to and inhibit Nek2A *in vitro*

**Fig. 7.**
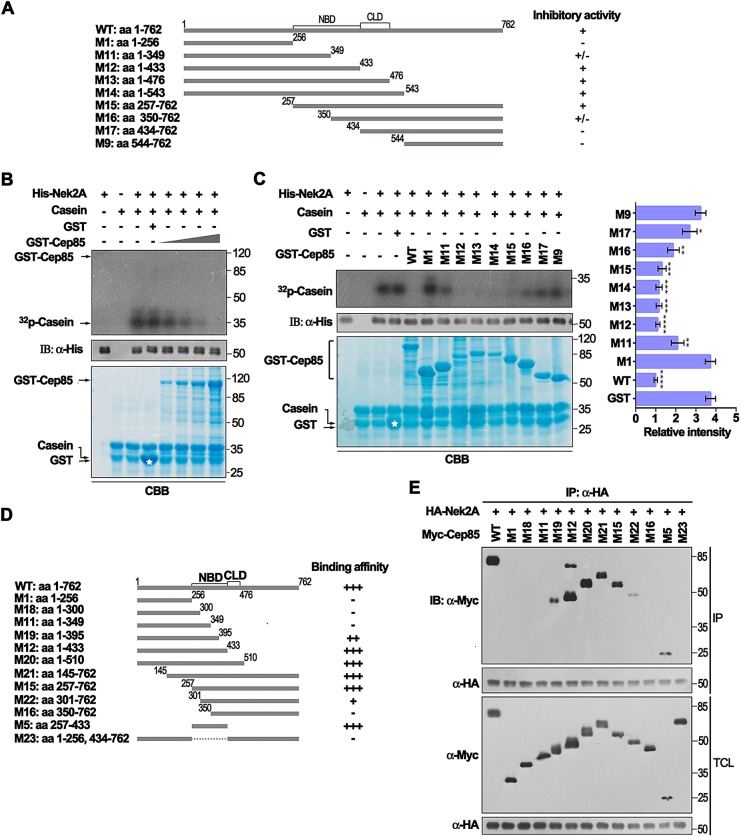
**Cep85 inhibits the kinase activity of Nek2A and the NBD in Cep85 is required for its activity.** (A) A schematic of Cep85 and its truncated mutants used in C are shown. The numbers indicate the positions of the first or the last amino acid of individual fragments. +, positive; +/−, partial; −, negative for inhibitory activity against Nek2A. (B) Cep85 inhibits the kinase activity of Nek2A in a dosage-dependent manner. The kinase activity of the purified recombinant His–Nek2A was determined using an *in vitro* kinase assays with β-casein as a substrate. The autoradiography shows [^32^P]β-casein. Purified recombinant GST–Cep85, GST protein and β-casein were visualized by Coomassie Brilliant Blue (CBB) stain. The asterisk indicates the position of GST protein. Nek2A levels were detected with an anti-His antibody (IB). (C) The NBD, amino acids 257–433 in Cep85, is essential for its inhibitory activity towards Nek2A. CBB stain was used to visualize GST–Cep85 proteins, β-casein and GST protein (marked by an asterisk). The autoradiography shows [^32^P]β-casein, and Nek2A levels were detected with anti-His antibody. The intensities of autoradiograph bands were quantified by densitometry and normalized to WT. The relative intensity was assessed and is shown on the right panel (mean±s.e.m., *n=*3). *P<0.05; **P<0.01; ***P<0.001; ****P<0.0001 versus GST. (D) The schematic of Cep85 truncated mutants used in E are shown. The numbers indicate the positions of the first or the last amino acid of individual fragments. +++, highest binding affinity to Nek2A; −, no binding to Nek2A. (E) The NBD, amino acids 257–433 in Cep85, is essential for its binding to Nek2A. Myc–Cep85 and its truncated mutants, overexpressed with HA–Nek2A in HEK293T cells, were immunoprecipitated (IP) and analyzed with western blotting.

Given that Cep85 can negatively regulate Nek2A activity in cells, we thus sought to determine the mechanism by which Cep85 could suppress Nek2A activity. We first examined whether Cep85 could inhibit Nek2A kinase activity. Nek2 kinase activity can be measured by an *in vitro* kinase assay with β-casein as a substrate (Fry et al., 1995). We found that GST–Cep85 could efficiently suppress Nek2A kinase activity in a dosage-dependent manner and that GST–Cep85 itself was not phosphorylated by Nek2A (Fig. 7B), suggesting that Cep85 is not be a substrate for Nek2A. We next determined which region in Cep85 was responsible for the inhibition of Nek2 kinase activity. A series of Cep85 truncated mutants were expressed and purified as GST fusion proteins (Fig. 7A), and Nek2A kinase activity was assessed in the presence of various Cep85 proteins. We found that those mutants harboring amino acids 257–433, including M12, M13, M14 and M15, retained full inhibitory activity towards Nek2A, similar to WT (Fig. 7A,C). Those mutants containing regions outside of amino acids 257–433, including M1, M17 and M9, failed to inhibit Nek2A; two additional mutants M11 (amino acids 1–349) and M16 (amino acids 350–762) containing part of NBD (amino acids 257–433) partially lost their activity to inhibit Nek2A (Fig. 7A,C). Thus, the region with amino acids 257–433 in Cep85 is responsible for suppressing Nek2A kinase activity.

To examine whether this region is able to bind to Nek2A, we subsequently generated Myc-tagged truncated mutant constructs of Cep85 (Fig. 7D) and carried out co-immunoprecipitation assays with HA–Nek2A. Comparing to WT, we observed that those mutants containing the intact region comprising amino acids 257–433, including M12, M15, M20 and M21, largely preserved their binding affinity to Nek2A (Fig. 7E). Those mutants containing a partial region of amino acids 257–433, such as M19 and M22, greatly lost their binding capacity to Nek2A (Fig. 7E). Remarkably, the mutant containing amino acids 257–433 alone fully retained the binding affinity, which was comparable to WT (Fig. 7E). In contrast, the internal deletion mutant M23, possessing all regions other than amino acids 257–433, completely lost its capability to bind to Nek2A (Fig. 7E). These results suggest that Cep85 binds to Nek2A through its internal region at amino acids 257–433 and inhibits Nek2A kinase activity. Thus, we defined the internal region with at amino acids 257–433 in Cep85 as a Nek2A-binding domain (NBD).

### Both the Nek2A binding and centrosomal localization domains are required for Cep85 function in cells

**Fig. 8.**
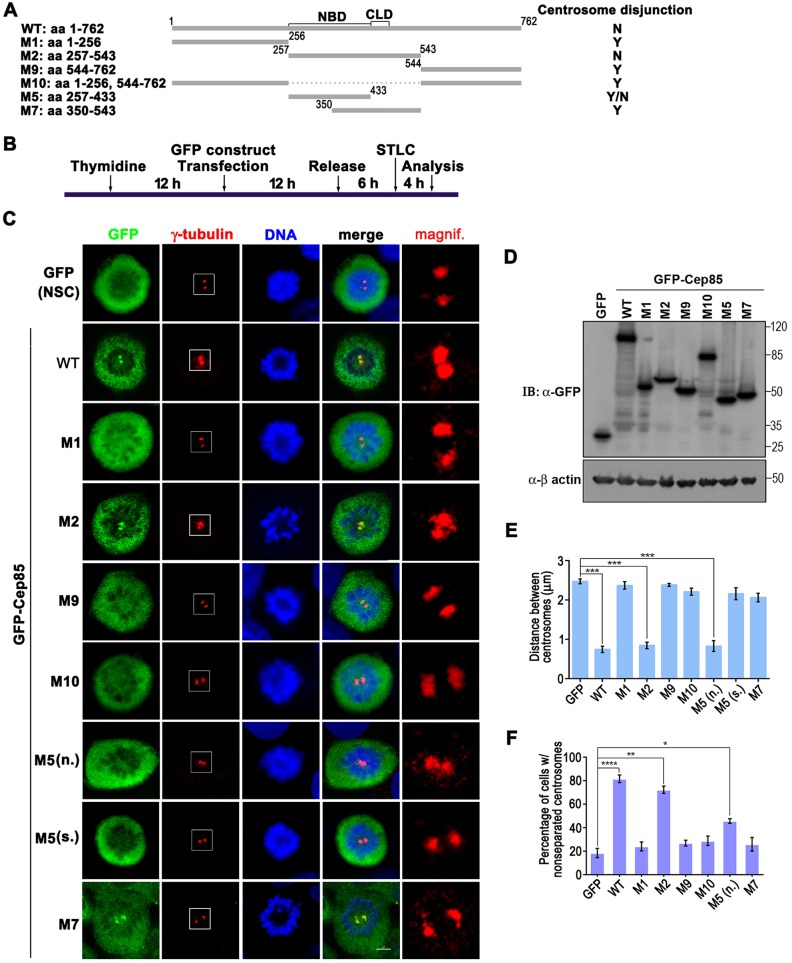
**Both the Nek2-binding and centrosome localization domains in Cep85 are required for its function in cells.** (A) A schematic of Cep85 and its truncated mutants used in C–F. The numbers indicate the positions of the first or the last amino acid of individual fragments. Y, yes; Y/N, partial; N, no for whether chromosome disjunction occurs. (B) Schematic outline of experiments in C–F. U2OS cells overexpressing GFP as a control (NSC, non-specific control) or the indicated GFP–Cep85 proteins were enriched at G2 by a single thymidine block and release, and treated with STLC to trap cells in the prometaphase prior to analysis. (C) Cells were fixed and stained for γ-tubulin (red) to indicate centrosomes. The boxed centrosomes are enlarged and are shown on the right. (D) The expression levels of GFP and GFP–Cep85 proteins in U2OS cells are shown by western blotting (IB) with anti-GFP antibody. (E) The distance between two centrosomes for cells in C. Results are from three independent experiments with 20 cells were analyzed for each experiment. Data are mean±s.e.m. ***P<0.001. (F) The histogram indicates the percentage of cell with nonseparated centrosomes. Centrosomes were considered separated when the *distance* between them exceeded 1 μm. Three independent experiments were performed with 20 cells were analyzed for each experiment. Data are mean±s.e.m. *P<0.05; **P<0.01; ****P<0.0001. M5(n.), M5 with nonseparated centrosomes; M5(s.), M5 with separated centrosomes.

Our *in vitro* kinase assay has clearly demonstrated that the NBD, located at amino acids 257–433, in Cep85 is sufficient to suppress Nek2 kinase activity and the centrosome localization domain at amino acids 434–476 together with the other regions are not essential for its inhibitory activity towards Nek2A (Fig. 7). We thus decided to examine whether the NBD region in Cep85 was also fully functional and suppressed centrosome disjunction in cells. Consistently, WT Cep85 when overexpressed could efficiently suppress centrosome disjunction (Fig. 8A–F); those truncated mutants not containing the NBD, including M1, M9 and M10, completely lost their capacity to suppress centrosome disjunction (Fig. 8). However, the mutant M5, which contains the NBD alone and retains the full inhibitory effect on Nek2 kinase activity *in vitro* (Fig. 7C), only partly prevented centrosome disjunction. Compared to those cells expressing WT Cep85, where ∼80% of them display nonseparated centrosome, only ∼40% of M5-expressed cells exhibited nonseparated centrosome (Fig. 8). In addition, the mutant M7, containing the centrosome localization domain and partial NBD, did not demonstrate any significant inhibitory activity (Fig. 8). Strikingly, the mutant M2 with these two domains joined together became fully functional (Fig. 8). The percentage of cells with nonseparated centrosome appeared to be ∼72%, close to that of WT (∼80%) (Fig. 8F). Thus, both the Nek2A-binding and centrosome localization domains are strictly required for Cep85 to efficiently suppress centrosome disjunction in cells.

## DISCUSSION

In current study, through affinity purification and mass spectrometry, we isolated and identified Cep85 as a binding partner of Nek2A. Further *in vitro* and *in vivo* studies confirmed the interaction of Cep85 and Nek2A. Moreover, immunofluorescence analysis revealed that endogenous Cep85 and Nek2A colocalize at centrosomes (Fig. 4). Importantly, Cep85 has been shown to localize to the proximal ends of centrioles, where Nek2A also localizes and functions as a kinase to phosphorylate the proteinaceous linker connecting two mother centrioles (Bahe et al., 2005; Fry et al., 1998a; Mardin and Schiebel, 2012). These findings reveal that Cep85 is a bona fide Nek2A-binding partner in cells.

To our surprise, at first glance, transient overexpression of Cep85 in both synchronized and unsynchronized cells did not cause significant defect in cell cycle progression. However, once treated with Eg5 inhibitor STLC, most Cep85-expressing cells arrested at prometaphase displayed centrosome disjunction failure and retained rootletin between two nonseparated centrosomes (Fig. 5). These results are exactly like those observed in cells with Nek2A depletion (Mardin et al., 2010). Similar results have also been shown by other treatments leading to reduction of Nek2A activity, such as inhibiting Plk1 using SB2536, inhibiting Aurora A with VX-680 and depletion of Mst1 and Mst2, or Sav1 (Mardin et al., 2011, 2010). By contrast, Cep85 depletion results in premature centrosome splitting and loss of rootletin at separated centrosomes in interphase (Fig. 6), strikingly reminiscent of Nek2A hyperactivation (Faragher and Fry, 2003; Fry et al., 1998b; Mardin et al., 2011). Importantly, co-overexpression of Cep85 with Nek2A could prevent the loss of rootletin at centrosomes caused by Nek2A overexpression in interphase (Fig. 5L). Thus, the results of the experiments with up- and down-regulation of Cep85 expression strongly suggest that Cep85 plays roles opposite to those of Nek2A in the maintenance of centrosome conjunction by suppressing Nek2A activity. Biochemically, we identified the region comprising amino acids 257–433 as the Nek2A-binding domain (NBD) in Cep85. Similar to WT Cep85, this domain is sufficient to bind to and inhibit Nek2A kinase activity (Fig. 7). We currently do not know exactly how Cep85 can suppress Nek2A kinase activity after it binds to Nek2A through the NBD. We noticed that Cep85, once co-expressed with Nek2A in HEK293T cells, can be phosphorylated (Fig. 1B), suggesting that Cep85 might be a substrate for Nek2A. However, in our *in vitro* kinase assays, we did not detect any incorporation of ^32^P into a GST–Cep85 protein (Fig. 7B), indicating that Cep85 might not be a substrate for Nek2A. Nevertheless, we found that Cep85 binds to the C-terminus of Nek2A outside of the kinase domain (Fig. 1), which has also been reported to be a pericentrin-binding domain. Whether this binding would cause any structural changes in Nek2A, as has been proposed for pericentrin (Matsuo et al., 2010), is certainly an interesting question and needs for further investigation. Interestingly, the NBD domain alone is not sufficient to efficiently prevent centrosome disjunction in cells, and an additional sequence carrying the centrosome localization domain (CLD) is also required for NBD to fully restore its activity (Fig. 8). Therefore, our data suggest a mode of Cep85 action in which it forms a granule meshwork enveloping the proximal ends of centrioles through its centrosome localization domain, where it interacts with Nek2A through its NBD, suppressing the kinase activity of Nek2A, thereby preventing centrosome splitting in interphase.

Nek2A has been shown to be the major kinase responsible for the phosphorylation and dissociation of the centrosomal linker proteins (Mardin and Schiebel, 2012). Its expression levels and kinase activity remain high in S to G2 phase (Hayes et al., 2006). Meanwhile, overexpression of Nek2A induces premature centrosome disjunction (Faragher and Fry, 2003; Fry et al., 1998b). Therefore, to ensure that the centrosome acts as a single MTOC in interphase, Nek2A kinase activity at centrosomes must be subject to strict control. Multiple levels of regulation have been reported to modulate Nek2A, including its expression, localization, and interaction with inhibitory proteins, like HEF1, pericentrin and PP1 (Fry, 2002; Mardin and Schiebel, 2012). For PP1, it has been demonstrated that PP1γ indirectly antagonizes Nek2A activity by dephosphorylating linker proteins rather than Nek2A *in vivo* (Mardin et al., 2011). Considering that the expression levels and activity of Nek2A are rather high throughout the S and G2 phase of cell cycle, this indirect suppression of Nek2A by PP1γ is certainly necessary but might be inefficient and uneconomic. In conjunction with the action of PP1γ, our model of Cep85 action provides a fairly efficient way to ensure that the two centrosomes will not be separated precociously. We propose that as Nek2A dynamically accumulates to the proximal ends of centrioles where Cep85 localizes, Cep85 binds to and traps it to form a kinase inactive Nek2A–Cep85 complex. The low amount of Nek2A protein that might escape from the trap would bind to and phosphorylate the linker proteins, such as C-Nap1 and rootletin. Immediately, its interacting protein PP1γ will then dephosphorylate them to prevent centrosome splitting in interphase. Although HEF1 and pericentrin might also be involved in restraining Nek2A activity in interphase, it has been suggested that HEF1 could counteract the positive regulation of Nek2A through two Hippo pathway components to prevent the accumulation of Nek2A at centrosomes (Mardin and Schiebel, 2012; Pugacheva and Golemis, 2005). Furthermore, given that neither HEF1 nor pericentrin has been shown to predominantly localize to the proximal ends of centrioles, they might not be heavily involved in the regulation of Nek2A activity there. As both Cep85 and PP1γ interact with Nek2A and reside at centrosomes at the proximal ends of centrioles at the right time, it is likely that our proposed mechanism is crucial for maintaining centrosome integrity in interphase.

In our studies, we have clearly demonstrated that Cep85 can suppress Nek2A activity. We have also found that Cep85 protein exists in all the cell lines we tested but the levels are different in our preliminary studies (Fig. 2). Given that Nek2A activity has been found to be elevated in many human tumors (Cappello et al., 2014; Hayward and Fry, 2006), it will be of great interest to examine the relevance of these two proteins by comparing the levels of Cep85 in primary cell lines with those in related cancer lines, as well as the levels in normal tissues with those in tumor samples, especially samples with high Nek2A activity.

## MATERIALS AND METHODS

### Plasmids and constructs

Human Cep85 coding sequence (NCBI accession number: NM_022778.3) was amplified from a cDNA library prepared by reverse transcription of total RNA isolated from HEK293T cells and subcloned into the mammalian expression vector pcDNA3.3-HA. This construct was verified by full-length sequencing and further subcloned into pcDNA-Myc, pEGFP-C3 and pGEX-4T-1 vectors. The truncated mutant constructs of Cep85 were prepared either by PCR amplification or subcloning using restriction enzymes with the parental construct pcDNA3.3-HA-Cep85 as a template. Nek2A (NCBI accession number: NM_002497) was cloned from HEK293T by RT-PCR and inserted into pcDNA3.3-Myc and pcDNA3.3-HA vectors. The kinase-dead Nek2A mutant (Nek2A K37R) was created by PCR-based site-directed mutagenesis as described previously (Fry et al., 1995). The truncated mutant Nek2A constructs were generated by PCR amplification. Nek2A was also subcloned into the donor vector pFastBac-HTA (Invitrogen) for expressing recombinant His-tagged Nek2A in insect cells. Centrin 1 was amplified from the HEK293T cDNA library and subcloned into peGFP-N1 for establishment of the centrin1–GFP stable cell line. Primer sequences used for PCR amplification and mutagenesis are available on request. All constructs were confirmed by sequencing.

### Cell culture, transfection, primary cilium induction and stable cell line establishment

Mouse fibroblast NIH 3T3, HEK 293T, human cervical cancer HeLa and human osteosarcoma U2OS cell lines were purchased from the ATCC. Cells were grown in high-glucose Dulbecco's modified Eagle's medium (Hyclone) plus 10% fetal bovine serum (Hyclone) and 100 IU/ml penicillin and 100 µg/ml streptomycin at 37°C in a humidified 5% CO_2_ incubator. Transient transfection of HEK293T was carried out with the cationic polymer polyethylenimine (PEI) according to the previous report (Boussif et al., 1995). TurboFect reagent (Thermo Scientific) was used to transfect HeLa and U2OS cells according to the manufacturer's instruction. To induce primary cilium formation, the growth medium was replaced with serum-free medium for NIH 3T3 cells for 24 h. To establish U2OS cell lines stably expressing centrin1–GFP, parental U2OS cells were transiently transfected with peGFP-N1-Centrin1 and selected with 50 µg/ml G418 for 2 weeks. Individual clones were picked up, multiplied and analyzed by fluorescence microscopy. Clones with a high signal-to-noise ratio were expanded for further analysis.

### Protein expression, immunoprecipitation and western blotting

Recombinant GST fusion Cep85 proteins were expressed in *E. coli* by transforming various pGEX-4T-1-Cep85 constructs into BL21 (DE3) bacteria, induced with 0.25 mM of IPTG, and affinity purified using glutathione–agarose resin (Thermo Scientific). Recombinant His–Nek2A protein was expressed in Sf9 insect cells using pFastBac-HTA-Nek2A as a donor plasmid according to the instruction manual of the Bac-To-Bac Baculovirus Expression System (Invitrogen) and affinity purified with Protino Ni-NTA Agarose (Macherey-Nagel).

For immunoprecipitation and western blotting, briefly, mammalian cells were lyzed in ice-cold cell lysis buffer containing 50 mM Tris-HCl pH 7.4, 150 mM NaCl, 1 mM EDTA and 0.5% NP-40, supplemented with 10 µg/ml pepstatin A, 10 µg/ml leupeptin and 1 mM PMSF. Cleaned cell lysates were incubated with antibodies at 4°C for 2 h prior to incubation with Protein A/G PLUS-Agarose (Santa Cruz Biotechnology) for an extra 2 h. The immunoprecipitates were washed, boiled in SDS sample buffer and resolved by SDS–PAGE. Western blotting was performed using standard protocols and the protein signal was visualized by chemiluminescence using the SuperSignal West Pico system (Thermo Scientific). β-actin was used as a loading control. Antibodies used included: rabbit anti-Cep85 (this study; 1:1000), mouse anti-Nek2A (BD Biosciences; 1:1000), mouse anti-Myc (SC-40, Santa Cruz Biotechnology; 1:1000), mouse anti-FLAG M2 antibody (F1804, Sigma-Aldrich; 1:500), mouse anti-HA (SC-7329, Santa Cruz Biotechnology; 1:1000), mouse anti-β-actin (AC15, Sigma-Aldrich; 1:5000), mouse anti-GST (26H1, Cell Signaling; 1:2000), mouse anti-cyclin B (GNS-1, BD Biosciences; 1:1000), rabbit anti-GFP (Ab290; Abcam; 1:5000) and mouse anti-His_6_ (ProteinTech; 1:5000) antibodies.

### siRNA

RNA oligonucleotides duplexes were synthesized by Shanghai GenePharma (Shanghai, China) and the sequences were as follows: Cep85, 5′-CCAACAGAACAAGUCCAUUtt-3′; Nek2A, 5′-AAACAUCGUUCGUUACUAUtt-3′; and negative control siRNA, 5′-UUCUCCGAACGUGUCACGUtt-3′. All siRNAs were transfected using ribo*FECT* CP (RiboBio, Guangzhou, China) and cells were analyzed 72 h after transfection.

### Cell synchronization

The double thymidine block was carried out as follows: cells were first incubated for 18 h with 2.5 mM thymidine followed by three quick washes with PBS and release in thymidine-free medium for 10 h. They were further incubated with 2.5 mM thymidine for an additional 16 h. Cells were washed three times with PBS, returned to fresh medium and allowed to grow for 2–16 h before harvesting the synchronized cells at different stages of cell cycle for analysis. To enrich cells at a specific stage, a single thymidine block was frequently used in this study. Cells were incubated in culture medium with 2.5 mM thymidine for 18–24 h, washed three times with PBS and then returned to fresh medium for hours. In some cases, 5 µM S-trityl-l-cysteine (STLC) was added to the medium 6 h after release to arrest cells at prometaphase.

### Immunofluorescence microscopy

For immunofluorescence, cells were fixed with ice-cold methanol for 5 min, blocked with 5% (v/v) bovine serum albumin (BSA) and 0.05% Triton X-100 in PBS for at least 30 min. Cells were subsequently incubated with primary antibodies with 5% (v/v) BSA and 0.05% Triton X-100 in PBS for 1 h followed by five washes with 0.1% Triton X-100 in PBS for a total of 30 min and another 1 h incubation with secondary antibodies. DNA was stained with DAPI (0.2 µg/ml; Invitrogen). Primary antibodies included rabbit anti-Cep85 (this study; 1:100), Alexa-Fluor-555-conjugated rabbit anti-Cep85 (this study; 1:10), mouse anti-γ-tubulin (T6557, Sigma-Aldrich; 1:2000), mouse anti-Nek2 (BD Biosciences; 1:200), rabbit anti-c-Myc (C-3956, Sigma-Aldrich; 1:400), rabbi anti-HA-tag (H6908, Sigma-Aldrich; 1:500), mouse anti-Sas-6 (SC-81431; 1:200), mouse anti-acetylated tubulin (T6793, Sigma-Aldrich; 1:500), rabbit anti-pericentrin (ab4448, Abcam; 1:3000), goat anti-rootletin (Santa Cruz Biotechnology; 1:500), rabbit anti-C-Nap1 (14498-1-AP, Proteintech, 1:1000). Secondary antibodies included donkey anti-mouse-IgG conjugated to Alexa Fluor 488, anti-rabbit-IgG conjugated to Alexa Fluor 488, anti-mouse-IgG conjugated to Alexa Fluor 555, anti-rabbit-IgG conjugated to Alexa Fluor 555 and anti-goat-IgG conjugated to Alexa Fluor 594 (Invitrogen). Images were acquired using a Zeiss LSM 780 laser-scanning confocal microscope (Carl Zeiss, Germany), or Axio Imager D2 equipped with AxioCam HRM (Carl Zeiss, Germany). Three-dimensional structured illumination microscope (3D-SIM) images were acquired on a DeltaVision OMX V4 microscope (Applied Precision, GE Healthcare, Issaquah, WA) equipped with a ×63, 1.42 NA Olympus objective. Data were reconstructed using API SoftWorx software.

### *In vitro* kinase assays

The *in vitro* Nek2A kinase activity measurement was performed as described previously (Fry et al., 1995). To examine the effects of Cep85 and its derivatives on Nek2A kinase activity, the recombinant Cep85 proteins were expressed and purified from *E. coli* and His–Nek2A was expressed and purified from Sf9 cells. The kinase reaction was carried out by incubating 0.02 mg/ml of His–Nek2A with ∼0.2 mg/ml of the GST-tagged Cep85 WT or truncated mutants in a total volume of 50 µl for 30 min at 30°C. GST protein was also included as a negative control. 0.5 mg/ml β-casein (Sigma-Aldrich, C-4032), as a substrate for Nek2A, was added to the kinase buffer containing 20 mM Tris-HCl pH 7.5, 50 mM KCl, 10 mM MgCl_2_, 1 mM DTT, 4 µM ATP and 10 µCi [γ-^32^P]ATP (Amersham). The amount of GST–Cep85 used for the experiment in Fig. 2B was 2 µg, 4 µg, 6 µg and 10 µg in a total volume of 50 µl. The reaction was terminated by adding 50 µl of 2× SDS sample buffer and boiling at 95°C for 10 min. The samples were solved by SDS-PAGE, stained with Coomassie Brilliant Blue (CBB) and subjected to autoradiography. His–Nek2A levels in the samples were visualized by western blotting.

### Measurement and statistics

ZEN 2010 software (Carl Zeiss, Germany) was used for measurement. The fluorescent intensity of Nek2A and γ-tubulin at centrosome and the distance of centrosomes were measured according to the methods previously described (Mardin et al., 2010). Statistical significance was determined by unpaired two-tailed Student's *t*-tests. Differences were considered as significant when *P*<0.05.

## Supplementary Material

Supplementary Material
