# Supplementary material for: Characterization of Cep85 – a new antagonist of Nek2A that is involved in the regulation of centrosome disjunction
Source: J Cell Sci. 2015 Sep 1;128(17):3290–303. doi: 10.1242/jcs.171637 (PMC4582193; doi:10.1242/jcs.171637)
Supplement: Supplementary Material [file supp_128_17_3290__index.html]

Characterization of Cep85 – a new antagonist of Nek2A that is involved in the regulation of centrosome disjunction — Supplementary Material 

# Characterization of Cep85 – a new antagonist of Nek2A that is involved in the regulation of centrosome disjunction

## JCS171637 Supplementary Material

- Supplementary Material
