# Supplementary material for: Characterization of Cep85 – a new antagonist of Nek2A that is involved in the regulation of centrosome disjunction
Source: J Cell Sci. 2015 Sep 1;128(17):3290–303. doi: 10.1242/jcs.171637 (PMC4582193; doi:10.1242/jcs.171637)
Supplement: Supplementary Material [file supp_jcs.171637_JCS171637supp.pdf]

Supplementary information

Supplemental figures

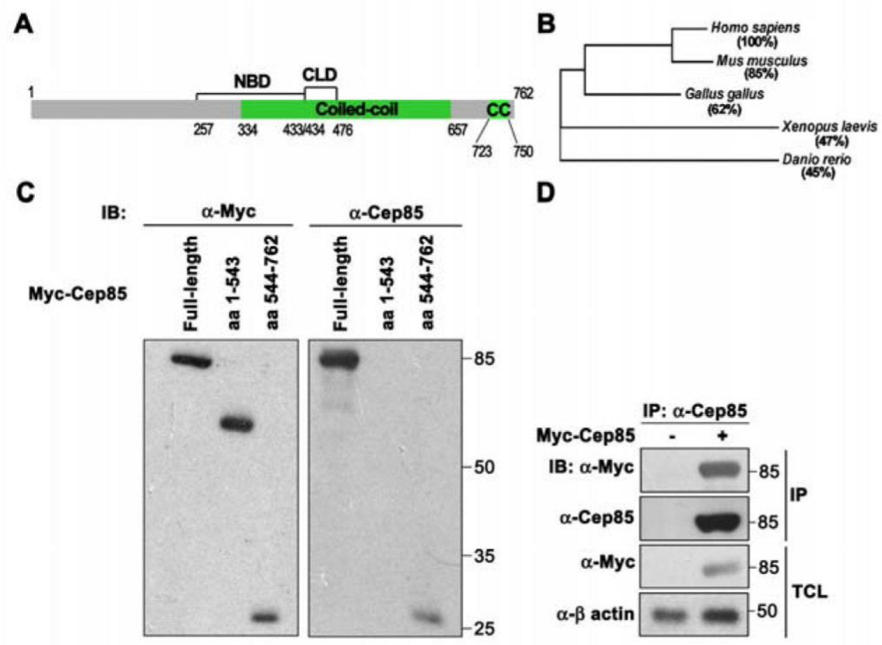

Fig. S1. Cep85 and rabbit polyclonal antibody anti-Cep85 (A) Schematic diagram of Cep85 protein. coiled-coil (CC) domains, Green: NBD (Nek2A binding domain, identified in this study), aa 257-433; CLD (centrosome localization domain, also identified in this study), aa 434-476. (B) Phylogenetic tree shows that Cep85 is a conserved protein. The percentages represent identities. (C-E) Characterization of Cep85 antibody. (C) Recombinant GST-Cep85 protein with the fragment of aa 544-762 was used as an antigen to immunize rabbits. The indicated Myc-tagged Cep85 proteins were expressed in HEK293T cells and the cell lysates were subjected to Western blotting with antibodies against Myc tag and Cep85. (D) The rabbit anti-Cep85 polyclonal antibody can be used for immunoprecipitation assays. Cell lysate from HEK293T cells overexpressing Myc-Cep85 was subjected to immunoprecipitation (IP) with anti-Cep85 antibody. Immunobotting with anti-Myc antibody recognizes the same spot detected by anti-Cep85 antibody. TCL, total cell lysate.

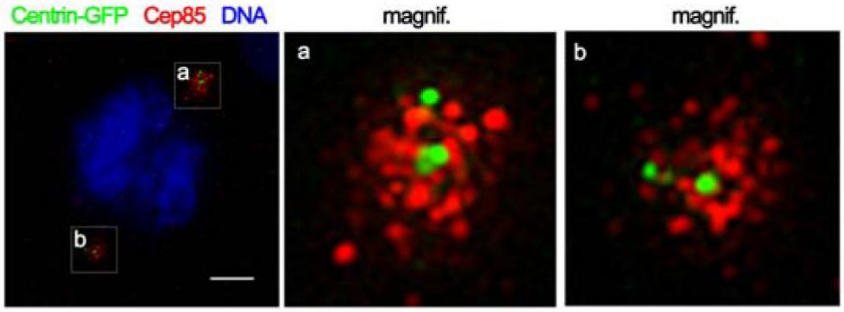

Fig. S2. 3D reconstruction of SIM images of Cep85 protein in a mitotic U2OS cell. The stable Centrin1-GFP U2OS cells were fixed and immunostained with anti-Cep85 antibody to unfold the structure of Cep85 (red) surrounding the centrioles and DAPI to stain DNA (blue). Scale bar represents 5 μm.

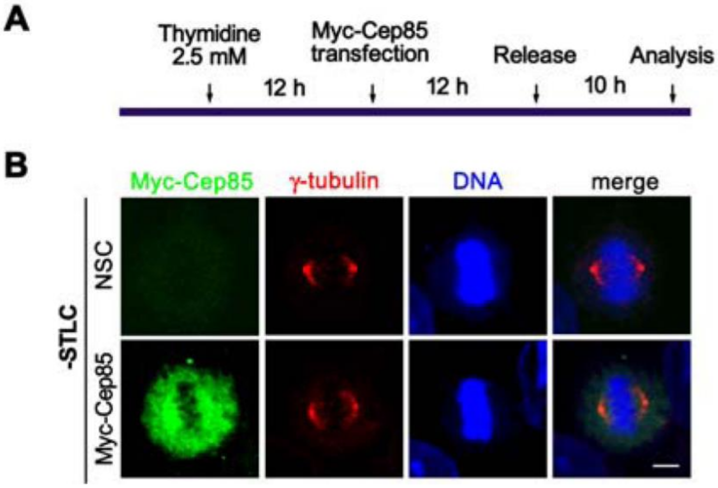

Fig. S3. Transient overexpression of Cep85 in U2OS cells does not arrest cells at specific cell cycle stages. (A) Schematic outline of experiments in (B). U2OS cells were treated essentially like those in Fig.5B except that they were not trapped with STLC at prometaphase. (B) Cells overexpressing myc-Cep85 were fixed and stained to visualize Myc-Cep85 (green), γ-tubulin (red) and DNA (blue) by immunofluorescence. Note: Cells overexpressing Myc-Cep85 can be found at different stages. Cells at metaphase with or without Myc-Cep85 are shown as representatives. Scale bar represents 5 μm.

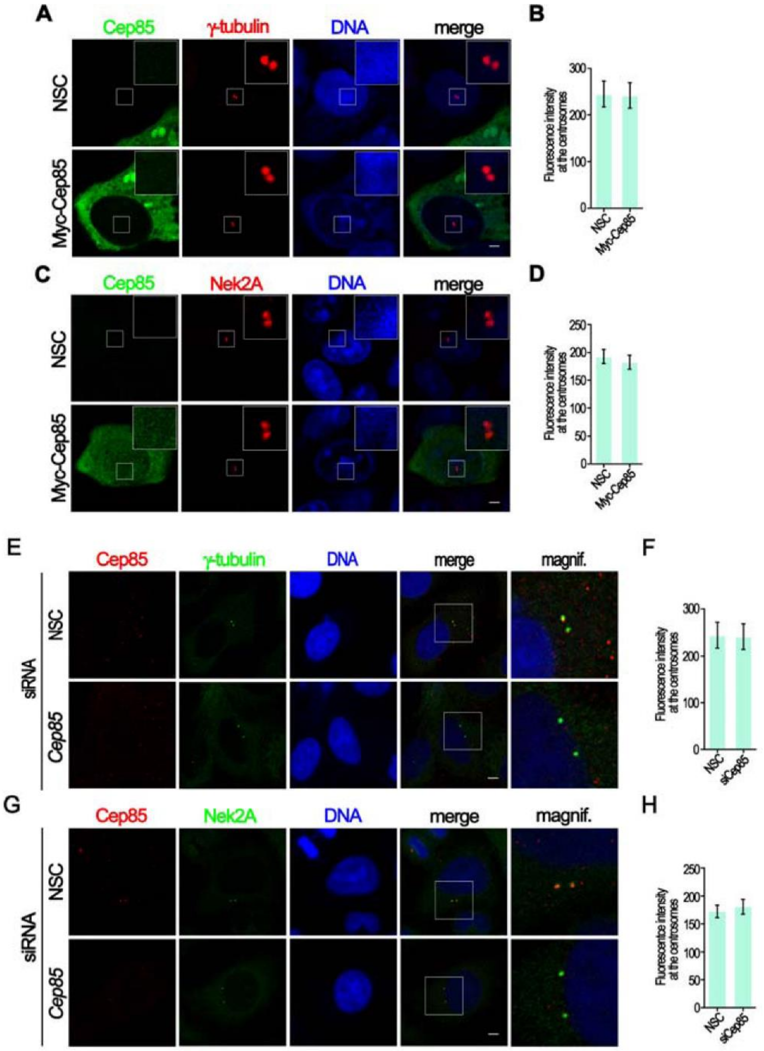

Fig. S4. Up- or down-regulation of Cep85 does not alter the intensity of γ-tubulin and Nek2A at centrosomes. U2OS cells overexpressing Myc-Cep85 were co-immunostained with anti-Myc antibody against Myc-Cep85 (green) and γ-tubulin (red) antibody in (A), and Nek2A (red) in (C). DNA (blue) was stained with DAPI. Cells at G2 selected as representatives are shown. Boxed areas are enlarged in insets shown on the top right. The intensity of γ-tubulin (B) and Nek2A (D) signals at centrosomes from cells in (A) and (C) , respectively, was quantified. Hela cells transfected with Cep85 siRNA or control siRNA were fixed 72 hr posttransfection and co-immunostained with anti-Cep85 antibody against endogenous Cep85 (green) and γ-tubulin (red) antibodyin (E), and Nek2A (red) in (G). DNA (blue) was stained with DAPI. Shown are cells at G2 selected as representatives. Boxed areas are enlargedand shown on the right. The intensity of γ-tubulin (F) and Nek2A (H) signals at centrosomes from cells in (E) and (G), respectively, was quantified. Data are mean ±s.e.m.. Results are from three independent experiments. 20 cells were analyzed for each experiment. Scale bars represent 5 μm.
